# Next-Generation Carbon-Based Quantum Dots for Healthcare and Beauty Applications

**DOI:** 10.3390/nano16030182

**Published:** 2026-01-29

**Authors:** Muhammad Noor Nordin, Nur Farhana Shahrul Azhar, Nurhakimah Norhashim, Ili Farhana Mohamad Ali Nasri, Noor Hafidzah Jabarullah

**Affiliations:** 1 Medical Section, British Malaysian Institute, Universiti Kuala Lumpur, Gombak 53100, Selangor, Malaysia; muhammadnoor@unikl.edu.my (M.N.N.);; 2 Avionics Section, Malaysian Institute of Aviation Technology, Universiti Kuala Lumpur, Dengkil 43800, Selangor, Malaysia; nurhakimah@unikl.edu.my (N.N.); nhafidzah@unikl.edu.my (N.H.J.)

**Keywords:** carbon quantum dots, green synthesis, cosmetic applications, wound healing, photoluminescence, sustainable nanomaterials

## Abstract

Carbon quantum dots (CQDs) have attracted intense research interest due to their unique physicochemical properties and broad application potential. CQDs are a new class of ultrasmall fluorescent carbon nanoparticles (<10 nm) that exhibit bright photoluminescence, broad excitation spectra, high quantum yields (QYs), and excellent photostability. Structurally, they consist of graphitic sp^2^/sp^3^-hybridized carbon with amorphous or nanocrystalline cores. Unlike conventional semiconductor quantum dots (SQDs), which often contain toxic group II–VI, III–VI, or IV–VI elements, CQDs offer a safer and more environmentally friendly alternative for biomedical and cosmetic applications. This review summarizes recent advances in green-chemistry approaches for CQD synthesis, including top-down, bottom-up, waste-derived, and surface-functionalization methods. Particular attention is given to natural carbon sources, which provide low-cost, sustainable, and eco-friendly routes for scalable production. The optical, electronic, and toxicological properties of CQDs are discussed to clarify their performance and safety profiles. Special emphasis is placed on their emerging roles in wound healing and cosmetic formulations, which remain underexplored despite their promising potential. To our knowledge, this is the first comprehensive review focusing on the current progress, key challenges, and future perspectives of CQDs in beauty and personal care applications.

## 1. Introduction

Owing to their high fluorescence, synthesis friendliness, water compatibility, and low toxicity, carbon quantum dots (CQDs) have been the most extensively researched carbon-based nanoscale materials over the last two decades [1,2]. Lead and cadmium heavy metal quantum dots have been used in semiconductor applications because of their high quantum efficiency and long-term oxidation resistance. Heavy metal quantum dots (QDs), however, are poisonous by nature and have complicated synthesis processes that can make them more expensive to create in large quantities [3]. In addition, depending on the degree of exposure to humans, heavy metals can cause chronic disease conditions such as brain damage, renal damage, anemia, and even death. Because the influence of light at a wavelength is longer than its emission wavelength, heavy metal QDs also exhibit reduced photobleaching resistance. Although non-toxic QDs, including zinc, indium, and silicon, have been suggested to address the toxicity issue [1] of semiconductor QDs, their high cost for mass production, owing to their complicated chemistry and poor efficiency [3], has become a concern. Carbon QDs (CQDs), a new class of fluorescent carbon-based nanomaterials with zero dimensions, offer advantages over existing carbon-based nanomaterials because they do not display toxicity [1,4,5,6].

When single-walled carbon nanotubes (CNTs), also known as carbon nanoparticles (CNPs), were purified in 2004 by Xu et al., CQDs were initially uncovered [7,8,9]. Since then, CQDs have gained attention owing to their unique characteristics such as excellent optoelectronic properties, photoluminescence (PL), straightforward synthetic routes, comparatively easy functionalization, low toxicity, good biocompatibility, and large surface area [10]. Compared to conventional fluorescent dyes, carbon dots display superior fluorescence, including photostability, resistance to photobleaching, and non-blinking, which is why they are known as fluorescent carbon [7,11]. Furthermore, they are desirable for technological applications because their optical properties can be tailored by controlling their size, chemical doping, and functionalization. Additionally, CQDs have other benefits, such as low cost and simple synthesis [3]. CQDs have been successfully applied in a variety of fields, including energy conversion and storage [12], multicolor light-emitting diodes (LEDs) [7,13,14], in vivo imaging [14], drug administration [2,7,14], fluorescence sensing [9], and cell imaging [7,13,14]. As a potential replacement for semiconductor quantum dots, CQDs have emerged as a research hotspot in the disciplines mentioned above [7,8,10]. Because of the different functional groups (-OH, -COOH, and -NH_2_) that have been added to the surfaces of CQDs through various chemical processes, they have a strong ability to connect with both organic and inorganic molecules. Chemiluminescence and electrochemical luminescence are produced by the remarkable electronic properties of CQDs (electron donor or electron acceptor, depending on the chemical structure). In optoelectronics, catalysis, and photovoltaics, CQDs are potentially useful [4]. CQDs typically have spherical, three-dimensional clusters with amorphous to nanocrystalline carbon cores smaller than 10 nm or 20 nm in diameter.

Most of the carbon atoms in the inner regions of the three-dimensional clusters are involved in sp^3^ hybridization, and a small amount is also involved in sp^2^ hybridization. CQDs’ crystal lattices are similar to those of graphite and amorphous carbon. The core–shell structure of CQDs is created through the nucleation process, which involves the steady development of the core and the formation of a functional group-filled shell that is “self-passivated”. According to a thorough investigation, the intrinsic states of the core can be either graphitic crystalline (sp^2^) or amorphous (mixed sp^2^/sp^3^), depending on the amount of sp^2^ carbon present. Most studies have reported tiny (2 to 3 nm) graphitic crystalline (sp^2^) cores with a typical lattice spacing of 0.2 nm.

The synthesis method, precursors employed, and other synthetic variables (temperature, time, and pH) all affect the type of core [4]. Based on the various carbon cores, CQDs are further separated into graphene quantum dots (GQDs), carbon nanodots (CNDs), and polymer dots (PDs). Similar to CQDs, GQDs are a type of OD nanomaterial in the graphene family that shares characteristics with graphene and carbon dots (CDs), which are anisotropic and typically have lateral dimensions of less than 20 nm. In contrast, CNDs are fluorescent carbon nanomaterials that can be made from a variety of carbon materials, including graphene, fullerene, graphite, and CNTs. The sizes of the molecules falling in the A class of CDs, known as PDs, are made up of a structure made of polymers or carbon hybrids that have a large number of surface carbon-based linkages and a large number of inner polymeric networks. Researchers continue to be puzzled by the PL mechanism of the CQDs. However, a widely recognized explanation includes the carbon-core state in CQDs and the surface state, molecule state, and quantum size impact. The quantum size effect states that the size of CQDs significantly affects their PL characteristics. The energy gaps and PL behaviors of different-sized CQDs differed significantly. The interaction between the carbon core and any connected surface functional groups is referred to as the surface state of the CQDs. The energy gaps of various surface functional groups vary, resulting in various emission sites. Consequently, it is possible to enhance the PL properties of CQDs by altering either the surface functional groups or the domain size of the sp^2^ conjugation. Their molecular states or PL centers emerge when the CQDs are produced at low reaction temperatures. The degree of carbonization increased with the consumption of the produced PL molecular moieties as the reaction temperature increased. These CQDs typically exhibit a high PL intensity and quantum yield (QY). The carbon-core state regulates the PL process when CQDs are created at high reaction temperatures. In contrast, the carbon-core state exhibits a weak PL characteristic and good photostability [2,15]. Green chemistry uses critical principles that minimize or avoid the application or synthesis of hazardous compounds in the development, production, and use of chemical products. When cleaner solvents are used, and CQDs are created from biowaste, they contain no or fewer harmful compounds compared to their predecessors, which results in more durable and affordable QDs.

CQDs are generally formed via top-down and bottom-up pathways. Most synthesis techniques require multiple steps, hard temperatures, and expensive carbon sources that may have hazardous effects; thus, green synthesis, specifically using a hydrothermal technique under a bottom-up approach, is preferable. The hydrothermal method can be used to produce CQDs with optical sizes ranging from 1 nm to 10 nm. In one study, spherical water-soluble CQDs (approximately 1 to 3 nm) were created from lemon peel waste using a cost-effective hydrothermal method, and the resulting stable carbon quantum dots were discovered to have excellent photoluminescence properties and oxygen-rich surface functionalities, with a quantum yield of approximately 14% [4].

The main motivation of this paper is to explain how different synthesis methods influence the structure, properties, and applications of carbon quantum dots (CQDs). In the past ten years, many researchers have developed both top-down and bottom-up techniques to improve the performance of CQDs. These approaches affect important material characteristics such as absorption, photoluminescence, and toxicity. This review brings together information on these synthesis routes, their related optical behavior, and the safety aspects of CQDs in a simple and organized way. It also highlights the increasing interest in using CQDs in health and beauty products, including applications for wound healing, anti-aging, skin regeneration, lip care, and sun protection. Through this review, the paper aims to give readers a better understanding of the potential of CQDs to support safe and sustainable developments in the biomedical and cosmetic fields.

## 2. Synthesis Method

**Figure 1:**
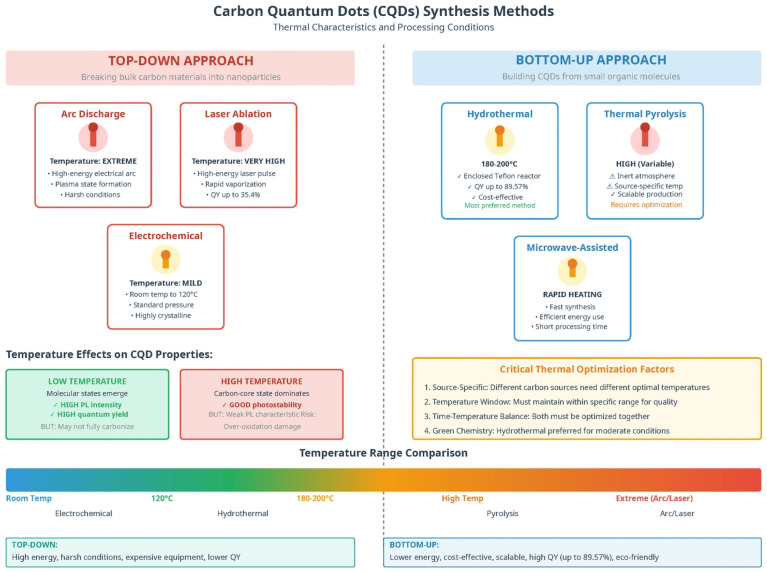
The synthesis of CQDs can be achieved using both top-down and bottom-up approaches. The conceptual illustration was prepared by the authors using AI-assisted design tools.

**Figure 2:**
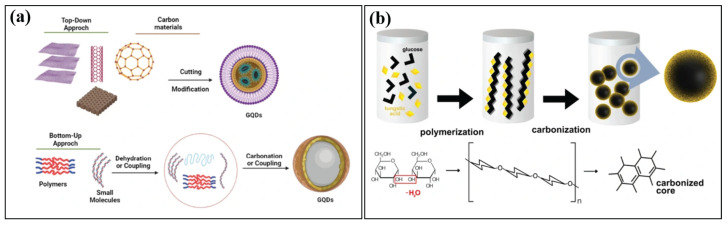
(**a**) The fluorescent GQDs were synthesized using two primary methods: the “top-down” splitting technique, whereby various carbon sources are used, and the “bottom-up” method, wherein small molecules or polymers are used as building blocks. Reproduced with permission from Ref [40]. Published by *Journal of the Brazilian Chemical Society*, 2023. (**b**) The steps from polymerization, carbonization, and passivation. Semiconductor nanocrystals, commonly known as quantum dots (QDs). Reproduced with permission from Ref [41]. Published by *Scientific Reports*, 2023.

In the past few decades, various synthetic techniques have been investigated and reported to enhance fluorescence by boosting the quantum yield and decreasing the bandgap, thus yielding longer wavelengths. They are synthesized in two ways (Figure 1): top-down and bottom-up. This production method may affect the sample size, functional groups, and stability of the colloidal suspension, directly affecting the physicochemical and optical characteristics of the CQDs [9,11]. Top-down methods have been utilized to convert numerous carbon sources into carbon powders, carbon nanotubes (CNTs), nanodiamonds, and other substances with excellent sp^2^ structures [15]. Some of the synthesis methods used in this study include electrochemical oxidation, chemical ablation, and arc discharge. Bottom-up CQDs are created from small carbonaceous molecules by a sequence of polymerization and carbonization events. Under this approach, CQDs can be produced using various synthesis methods, including hydrothermal, thermal pyrolysis, and microwave-assisted methods. The top-down strategy used severe conditions, expensive materials, and a time-consuming process, making the bottom-up approach more advantageous (Figure 2).

### 2.1. Top-Down Approach

The advantages of employing a top-down technique are its simplicity, speed, and versatility for producing different types of nanostructures. In top-down procedures, bulk carbon materials (such as large-sized graphene, CNT, graphite, and commercial activated carbon) are chemically or physically chopped into CQDs before being used to create carbon dots (Figure 2a). Arc-discharge [16,17,18], laser ablation [19,20,21,22], electrochemical [23], plasma reactor, ultrasonic synthesis, chemical exfoliation, and combustion procedures are some of the techniques used in these processes. However, in contrast to bottom-up approaches, the synthesis of CQDs using these techniques is typically carried out under rigorous experimental conditions, with onerous operation steps and expensive equipment being typically employed, which significantly restricts their practical application [24].

#### 2.1.1. Arc-Discharge Technique

Arc discharge is the most popular and high-energy method for generating bulk carbon raw materials. Xu et al. used arc-discharge techniques to create three different types of carbon nanoparticles with various fluorescence and relative molecular mass characteristics. They produced a potent black slurry by extracting the chamber with NaOH and oxidizing arc-discharge soot with 3.3 N nitric acid to introduce the -COOH groups [16,25,26]. This approach results in CQDs with high oxygen concentrations and natural fluorescence characteristics. According to Liu et al., a DC arc charge can produce amorphous CNT. Additionally, the detection of nanoparticles in the carbon arc-discharge technique with laser-induced incandescence was performed when Yatom et al. identified nanoparticles with minimal diameters of 500 nm. The size diminishes as one moves away from the arc’s axis [17]. However, it is difficult to purify these segments due to the large number of composite segments employed in this method [24].

#### 2.1.2. Laser Ablation Technique

In laser ablation procedures, the surface is irradiated with a high-energy laser pulse and a carbon source to increase the temperature and pressure before the surface crystallizes into nanoparticles (NPs). The target molecules are quickly vaporized by this and moved into a plasma state, where they condense to create nanoparticles. In their research, Sun et al. [19] used dual-beam pulsed ablation to create uniform and stable CQDs from inexpensive carbon fabric. The high-temperature pyrolysis of the surface of the carbon fabric creates substituents that contain oxygen and sulfur. The related quantum yield of emission for PL emission and cell bioimaging can reach 35.4%, which is higher than the yield for the laser ablation of CQDs by a single-beam pulse. In HeLa cell bioimaging, CQDs with outstanding stability and excellent anti-jamming performance were used and exhibited good biocompatibility [27]. After being synthesized by a practical and straightforward method using double-pulse femtosecond laser ablation in solution, CQDs exhibit ultrasmall diameters and an abundance of surface functional groups [21]. The laser ablation approach is a cutting-edge and efficient method to create CQDs, but its use is limited by the cost and complexity of the procedure [25].

#### 2.1.3. Electrochemical Technique

Electrochemical approaches are the most well-liked, straightforward, speedy, economically practical, highly productive, and reproducible methods for creating CQDs from bulk materials, such as graphene, graphite, and carbon fiber. This method produces highly crystalline CQDs with a highly crystalline and uniform size distribution at standard pressure and temperature levels. Diverse electrode materials are used in electrolytic solutions to carry out electrochemical oxidation or reduction processes. By adjusting the applied voltage, the CD size could be tailored electrochemically. Zhou et al. first demonstrated this technology, which was then used for chemical analysis and bio-related applications [28]. Pang et al. collected CQDs during reflux after dissolving the oxidized carbon fiber in concentrated nitric acid and continued the reflux process at 120 °C [14]. On the other hand, by electrochemically etching carbon fibers, Bao et al. [23] have created a controllable approach to create luminous C-dots with a tiny size and long emission wavelength.

### 2.2. Bottom-Up Approach

In the bottom-up method, organic compounds such as citric acid and saccharides are typically used as building blocks for the synthesis of CQDs (Figure 2a). In addition, by altering the experimental conditions, a bottom-up technique can cost-effectively and scalably generate CQDs of the desired size. Hydrothermal and thermal pyrolysis are the most frequently employed bottom-up approaches [14]. Regardless of the methods used to manufacture them, the carbon dots have a variety of sizes, necessitating the use of sophisticated separation processes to produce monodispersed carbon dots [15]. The other bottom-up approaches involve the use of microwaves [10,29,30], pyrolysis [31,32,33], solvothermal methods [34,35,36], and ultrasonic methods [37,38,39], which, on the other hand, use carbonization and passivation to transform tiny molecules into carbon dots. The benefits include low costs, simple equipment requirements, and easy operation; as a result, they are frequently used in the synthesis of carbon dots [2].

#### 2.2.1. Hydrothermal Technique

Synthesis of CQDs from saccharides or organic acids is an easy and affordable process. This method creates CQDs by carbonizing organic precursors at a high temperature (180–200 °C) in a hydrothermal reactor that is enclosed and lined with Teflon. According to Figure 2b, Hsu and Chang found that the hydrothermal and development model of CQDs consists of four steps: dehydration, polymerization, carbonization, and passivation [42]. A frequently employed technique for creating unique carbonic nanomaterials from a range of fundamental raw materials is hydrothermal carbonization. This is because hydrothermal carbonization is typically a simple, inexpensive, environmentally friendly, photobleaching-resistant, and non-toxic one-step procedure. This generates CQDs with high brightness and virtually uniform size [43]. The amino and carboxylic acid groups are helpful in the synthesis of CQDs. In the hydrothermal approach, hydrogen bonding facilitates molecular assembly and heating to promote CQD development, polymerization, and nucleation. Precursors, such as glucose and monopotassium phosphate (KH_2_PO_4_), were used in a hydrothermal method to create CQDs [12]. By adjusting the nitrogen content during the hydrothermal decomposition of citric acid and urea, Hola et al. created tunable fluorescent full-color CQDs [44]. Additionally, by adjusting the temperature while using biomolecules high in carbon and nitrogen, 89.57% of the QY may be produced in CQDs under hydrothermal processes [45]. This approach is beneficial for bioimaging and biosensors because it is straightforward and capable of regulating N-doped CQDs [9].

#### 2.2.2. Thermal Pyrolysis Technique

Pyrolysis is the standard method for creating carbon dots. Carbon dots are created by heating, dehydration, degradation, and carbonization of the organic material in the carbon source at high temperatures in either a vacuum or an inert atmosphere. The pyrolysis process often employs high concentrations of acids or alkalis to break the carbon precursors into nanoparticles. For the pyrolysis process to create carbon dots, various biomass materials, such as watermelon peel, sago waste, coffee grounds, and plant leaves, can be used as carbon sources. By adjusting experimental parameters such as reaction temperature, reaction time, and pH of the reaction system, the physicochemical and optical properties of the resulting carbon dots can be tailored [46]. Zhou et al. produced carbon dots on a wide scale by pyrolyzing watermelon peels at low temperatures, followed by filtering [47]. The carbon dots exhibit strong blue luminescence, excellent water solubility, and outstanding stability in solutions with a wide range of pH and high salinity [2]. The pyrolysis method has been widely employed to create CQDs through carbonization of organic molecule-based precursors at a higher temperature in an inert atmosphere. Precursors participate in a number of processes throughout the carbonization process, including dehydration, polymerization, carbonization, and creation of carbon dots. This approach has the following benefits: it is quick, easy, and cheap, and it reduces water pollution and greenhouse gas emissions [11]. Giannelis et al. first demonstrated this method for synthesizing CQDs and used thermal carbonization to create CQDs with surface functionalization from various ammonium citrate salts. The same group revealed how carbogenic CQDs were produced by pyrolyzing ethanolamine (EA) and CA at various temperatures, demonstrating the significant impact of temperature on the PL behavior of CQDs during synthesis.

The preparation of CQDs from readily available, low-cost materials and their encapsulation with biocompatible polymers, which are important for biomedical applications, including bimodal imaging and drug delivery, are affordable and scalable methods [15]. Dialysis must be used to cleanly separate the CQDs produced by this method. By adjusting the experimental parameters, this method can be used to create monodispersed CQDs with an average particle size of less than 10 nm [48]. One of its main advantages lies in its simplicity and scalability. Pyrolysis is typically a one-step thermal decomposition process that can be readily adapted for continuous or batch operations [49]. This process requires minimal equipment and can easily be scaled from laboratory to industrial levels without significant modifications to the setup [49].

In terms of cost-effectiveness, pyrolysis synthesis stands out for its use of low-cost and abundant precursors, such as organic compounds, polymers, or even biomass-derived materials [50]. This results in reduced material and operational costs compared to multi-step or solvent-intensive synthesis routes. Moreover, the technique’s thermal decomposition nature often eliminates the need for expensive catalysts or complex purification procedures, further improving its affordability and suitability for upscaling.

Beyond its general advantages, the choice of precursors, particularly organic-based materials, plays a pivotal role in determining the properties of the final nanomaterials. Organic precursors such as lignin, cellulose, starch, and various polymers are increasingly favored due to their abundance, renewability, and carbon-rich structures. For instance, pyrolytic lignin has been identified as a promising green precursor for producing fluorescent carbon nanoparticles under aqueous pyrolysis conditions, offering both sustainability and high optical performance [9]. Similarly, cellulose nanocrystals derived from agricultural waste (e.g., oil palm empty fruit bunches) have been successfully converted into carbon nanodots, where the pyrolysis temperature dictates particle size and fluorescence intensity [8]. Starch-based precursors have also demonstrated potential in electrochemical applications, serving as cost-effective carbon sources for lithium-ion battery anodes with tunable porosity and conductivity depending on carbonization conditions [22]. Table 1 summarizes the CQDs’ synthesis methods and easy operation; as a result, they are frequently used in the synthesis of carbon dots [2].

### 2.3. Functionalization

All carbon dots feature chemical groups, such as oxygen- or amino-based groups and polymer chains, on their surfaces, and these functional groups are crucial to photoluminescence behavior. Because of the * transition of the C = C bonds, carbon and graphene quantum dots are equally effective in photon-harvesting in the short-wavelength region. In general, CQDs have a low quantum yield and are considerably more efficient at absorbing long wavelengths, necessitating the addition of a surface passivation layer to increase brightness. Due to their layered structure and superior crystallinity, graphene quantum dots often yield higher quantum yields than carbon dots. Carbon dots of various hues, spanning from ultraviolet to red and, frequently, blue and green, have been created. The considerable heterogeneity (in size and chemical composition) resulting from poorly regulated manufacturing techniques makes most of these carbon dots undesirable for multicolor imaging and causes them to exhibit broad emission spectra [13]. Because the addition of functional groups, such as amines and carboxyl, can impose various flaws on the CQD surface, the functionalization of CQDs is crucial. Fluorescence emissions are incredibly variable because these imperfections act as excitation energy traps. Adding carbonyl and carboxyl groups to the surface of CQDs through oxidative treatment with strong acids is an easy and very successful way to increase the water solubility of CQDs. To create CQDs, an oxidative acid, such as nitric acid, is frequently added to the reaction mixture [3]. Liu et al. demonstrated that an oxidant is required to generate visible fluorescence emissions. Their team suggested that this acid oxidative treatment might also be used to disintegrate larger carbon clumps into nanoparticles.

### 2.4. Major Factors Affecting the Properties of C-Dots

#### 2.4.1. Raw Materials

The raw materials utilized during the carbon dot production of CDs may affect the fluorescence characteristics of the CQDs. For instance, when the same synthesis technique is applied to cucumber and pineapple peels, there are significant differences in the properties of the carbon dots. For instance, CDQs obtained from the pineapple peels were completely degraded, whereas the CDQs obtained from the cucumber peels maintained good stability. In addition, fungal growth was observed on the surfaces of pineapple CQDs but not on cucumber peels. Therefore, CQDs made from cucumber peels are promising for use in organic electronics and bioimaging. Using the same synthetic method, Boruah discovered that CQDs made from sugarcane bagasse, garlic peels, and taro peels had different quantum yields (4.5% and 13.8%) [2].

#### 2.4.2. Synthesis Temperature

A carbonization procedure was used to create fluorescent CQDs using biomass waste as the carbon source. Temperature plays a crucial role in the synthesis of CQDs because carbonization is an endothermic process. Using an easy and affordable pyrolysis technique, Zhu et al. studied the effect of the pyrolysis temperature on the luminescence characteristics of C-dots created from various plant leaves, including lotus leaves, pine needles, oriental plane leaves, and palm leaves. According to their experimental findings, different carbon sources require varied optimum pyrolysis temperatures when creating CQDs. Therefore, it is essential to maintain the pyrolysis temperature within a specific range. The carbon source may not completely carbonize the CQDs if the pyrolysis temperature is too low. Although the surface structure of CQDs is broken, the carbon source over-oxidizes at very high temperatures, resulting in a decline in the optical performance of the CQDs [2].

#### 2.4.3. Reaction Time

The reaction temperature and duration both affect the optical characteristics of the CQDs. The excessive carbonization that results from the slow reaction time destroys the surface structure of the CQDs. However, a quick reaction time will result in low carbonization of the carbon source, leading to CQDs with weak fluorescence emission. It should be noted that the response time has a temperature-dependent impact on the optical characteristics of CQDs. Optimization of the reaction time has any value only when the reaction is conducted at the appropriate temperature. Even if a lengthy reaction time is used, a usable end product can only be produced at a sufficient reaction temperature [2].

#### 2.4.4. pH Value

The effect of the solution pH on the fluorescence emission intensities of CQDs made from various carbon sources using various synthetic methods varies. Some CQDs favor neutral pH, some perform well under acidic conditions, and some can only function correctly in alkaline environments, whereas others are stable across a wide pH range. Since the fluorescence emission intensities of CQDs produced from biomass waste change in response to a change in pH value, they can be employed as pH sensors [2].

#### 2.4.5. Surface Passivation

In general, unmodified bare CQDs have a poor fluorescence quantum yield. Surface passivation or functionalization is frequently utilized to increase the fluorescence emission intensity of CQDs and hence increase their application in bioanalytical tests. CQDs’ surface passivation can reduce surface flaws and boost the likelihood of exciton–hole recombination, preventing CQDs from clumping together and increasing the intensity of their fluorescence emission. Two main surface passivation methods are coating bare CQDs with long-chain agents and oxidizing the surfaces of CQDs with potent acids [2].

## 3. Optical Properties

The optical features of QDs have attracted much interest and excitement in various applications, including the health and beauty industries, due to their broad excitation and emission wavelength ranges. The CQDs have been characterized using various methods, including UV-Vis-NIR absorption, photoluminescence (PL) spectroscopy, and cytotoxicity.

### 3.1. UV-Vis-NIR Absorption

**Figure 3:**
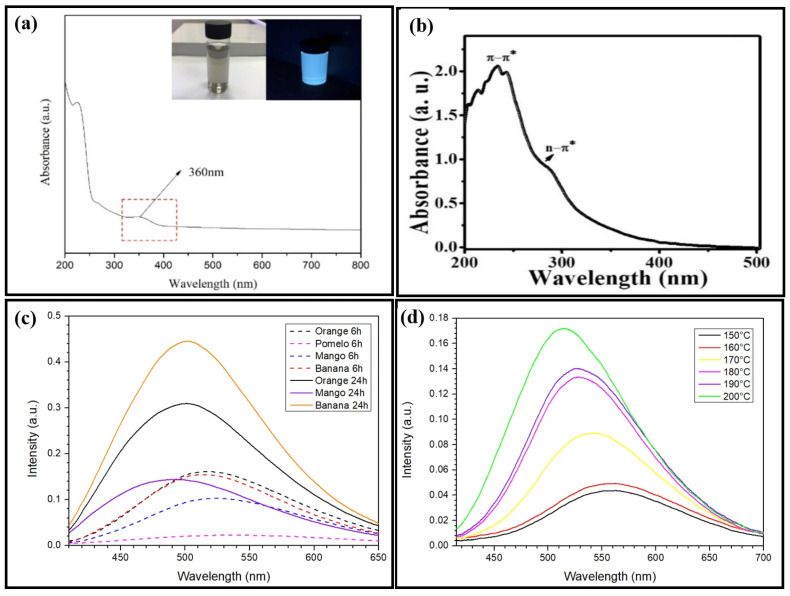
UV-Vis absorption spectrum of (**a**) empty fruit bunch biochar CQDs. Reproduced with permission from Ref. [51]. Published by *Materials*, 2020. (**b**) Natural lemon juice CQDs. Reproduced with permission from Ref. [54]. Published by *Journal of Advanced Biomedical Sciences*, 2022. Experimental results of the fluorescence emission spectra of the corresponding CQDs from (**c**) orange peels, mango peels, and banana peels undergo a hydrothermal synthesis route for a duration between 6 and 24 h; (**d**) banana peels synthesized under different hydrothermal temperatures (150–200 °C).

UV-Vis-NIR absorption is used to measure the amount of photon energy required for an electron to transition from its ground state to its excited state. CQDs exhibit absorption in both the UV region (230 to 280 nm) and the visible light region (400 to 650 nm). The absorption peak at a lower wavelength indicates the -* transition of the carbogenic core. In contrast, the C=O and C=N bonds of the n-* transitions of the surface functional groups are responsible for the higher wavelength peak. Previous research found that the absorption spectrum of empty fruit bunch biochar CQDs (Figure 3a) produced two peaks at 226 nm, attributed to the sp^2^-conjugated C=C bond’s -* transition and 360 nm as a shoulder absorption peak related to the C=O bond’s n-* transition [51]. In contrast, it has been reported that the absorption peak is detected at 254 nm for durian shell CQDs, with absorption shoulder peaks occurring at 280 nm and 320 nm [52]. According to the authors, their sample of banana peel CQDs produced two absorption peaks in the UV area at 277 nm and 322 nm, which are comparable to those of the empty fruit bunch biochar CQDs and are associated with the typical -* and n-* transition bands [53]. Although these earlier studies employed the same hydrothermal method, the location of the absorption peak was different because the CQDs were produced using different primary materials at different temperatures and over a longer period of time. The natural lemon juice CQDs exhibited two clear absorption peaks around 232 nm and 283 nm (Figure 3b), which are interpreted as the specific bonds of typical -* and n-* transition bands. These characteristic absorption features confirm the successful formation of CQDs from the natural lemon juice [54]. According to Wang et al. [25], adding 0.5g of anhydrous citrus extract to a solution of N-(aminoethyl)-aminopropyl methyl dimethoxy silane results in a prominent UV–visible absorption peak at 360 nm. Citrus acid was carbonated by Dong et al. to create CQDs that were between 15 and 0.5 nm wide and between 0.5 and 2.0 nm thick, displaying UV absorption at 362 nm and 460 nm in the absorption range [55]. Tang et al. stated that an aqueous solution of these C-dots exhibited two apparent UV absorption peaks at 228 and 282 nm by pyrolysis of a glucose solution assisted by microwaves for the manufacture of CQDs [56]. While the peak positions remained unchanged, increasing the microwave heating duration enhanced the strength of both the UV absorption peaks. Strong UV absorption can be observed while fabricating CQDs using various synthesis methods; however, the peak UV absorption sites vary according to the method.

### 3.2. Photoluminescence (PL) Spectroscopy

The most notable characteristic of CQDs is their tunable photoluminescence, which gives them a broad spectral range and high-intensity emission peaks in the ultraviolet, visible, and NIR regions. This essential characteristic is also known as the excitation-dependent luminescence spectra. The quantum confinement effect (QCE) is thus used to control the emission color of CQDs according to the supplied excitation wavelength, and the size of the nanoparticles illustrates the excitation-dependent luminescence spectra of a CQD sample and their corresponding colors. The surface trap state is known to cause bimodal Gaussian peaks in conventional QDs; however, research on the surface trap states that distinct energy levels in quantum wells (CQDs) are still in their infancy and need to be expanded.

CQDs of various sizes emit light of varying hues and intensities. For our preliminary experiment, the synthesis time was manipulated to observe the photoluminescence of the CQD sample. The synthesis temperature is fixed at 200 °C. Orange peels, mango peels, and banana peels undergo hydrothermal synthesis for durations between 6 and 24 h (Figure 3c). Based on our results in Figure 3d, the PL intensity of banana peel CQDs becomes greater with increasing synthesis temperature. According to reports, empty fruit bunch biochar CQDs exhibit blue-green color samples under UV light and a grayish color under natural light with a 425 nm PL intensity [51]. Similarly, durian shell and banana peel CQDs displayed vivid blue under UV light and yellow under natural light, with PL intensities of 414 nm and 365 nm, respectively [52,53]. By altering the input laser in the laser ablation approach, Yu et al. discovered variable-size CQDs, which demonstrated size-dependent fluorescence emission in the visible area due to the quantum confinement effect [57]. Additionally, Peng et al. created several fluorescence emissive carbon dots in blue, green, and yellow by altering their sizes (1 to 4 nm, 4 to 8 nm, and 7.11 nm), which changed the reaction temperature [58]. When activated by longer wavelength light from 500 to 1000 nm, Li et al. exposed CQDs and emitted light in the range of 325 to 425 nm [59]. These findings suggest that the quantum-sized graphite structures of the CQDs were responsible for their unique up-conversion phenomenon and size-dependent fluorescence emission capability. An up-conversion event is typically a form of the emission process. This substance uses successive multiphoton absorption to produce light emission at a wavelength shorter than the excitation wavelength.

#### Modeling for Size-Dependent Optical Properties of CQDs

The modeling of size-dependent optical properties in colloidal quantum dots (CQDs) requires a comprehensive understanding of quantum confinement effects and their manifestations in the electronic and optical properties of these nanoscale materials. When the physical dimensions of a semiconductor nanocrystal become comparable to or smaller than the exciton Bohr radius, the charge carriers experience quantum confinement, which fundamentally alters the electronic structure and optical properties compared to the bulk material [60,61]. The equation is

(1) $$E_{core}\left(R\right)=E_{bulk}+\frac{\hbar^{2}\pi^{2}}{2R^{2}}\left(\frac{1}{m_{e}^{*}}+\frac{1}{m_{h}^{*}}\right)-\frac{1.8e^{2}}{4\pi \varepsilon_{0}\varepsilon_{r}R}$$

where the $E_{bulk}$ is the effective bulk energy gap of the carbon core, $m_{e\;}^{*}$ and $m_{h\;}^{*}\;$ are the electron and hole effective masses, $\varepsilon_{r}$ is the relative dielectric constant, *ℏ* is the Planck constant, and *e* is the elementary charge. The second term represents the quantum confinement energy shift, while the third term accounts for the Coulomb attraction between charge carriers. This equation predicts that the bandgap widens as the CQD size decreases, resulting in higher PL emission energy (shorter wavelength). Although CQDs are partially amorphous and sp^2^–sp^3^-hybridized, the model provides a useful phenomenological description of the size–emission relationship [60,61]. In addition to core-state emission, surface functional groups (e.g., -OH, -COOH, and -NH_2_) introduce localized energy states that influence optical behavior. The surface contribution can be represented as

(2) $$\omega_{surf}\left(R\right)=A_{surf}\frac{S}{V}=A_{surf}\frac{3}{R}\;$$

where $\omega_{surf}$(*R*) is the weighing factor for surface emission, $A_{surf}$ is an empirical constant, *S* is the surface, *V* is the volume and *R* is total emission intensity. This expression reflects that smaller CQDs, with larger surface-to-volume ratios, exhibit stronger surface-state luminescence. Consequently, the total emission intensity can be treated as a sum of core and surface components:

(3) $$I_{total}\left(E\right)=I_{core}\left(E\right)+I_{surf}(E)$$

where *E* represents the photon emission energy. Because CQDs are typically synthesized as ensembles with a range of particle sizes, the observed PL spectrum represents a convolution of emissions from all particles:

(4) $$I\left(E\right)=\;\int_{0}^{\infty }P\left(R\right)\left[I_{core}\left(R\right)g\left(E;E_{core}\left(R\right),\;\sigma_{core}\right)+I_{surf}\left(R\right)g\;\left(E;E_{surf}(R),\sigma_{surf}\right)\right]\;dR$$

$P\left(R\right)$ is the particle size distribution (often log-normal), and $g(E;E_{i},\sigma_{i})$ denotes the Gaussian lineshape function centered at $E_{i}$ with width $\sigma_{i}$. This ensemble model allows simulation of experimental PL spectra based on measured size distributions from TEM or AFM.

### 3.3. Cytotoxicity

Cytotoxicity assays can be used to assess the quality of CQDs for cell bioimaging because biocompatibility and bioavailability are important factors that must be considered. According to ISO 109993-5, cytotoxicity assays include extraction, direct contact, and indirect contact tests. It is a biological evaluation and screening test that uses in vitro tissue cells to observe the effects of nanomaterials on cell growth, reproduction, and morphology [62]. Because it is easy, quick, sensitive, and can protect animals from toxicity, cytotoxicity is preferred as an important signal for the toxicity evaluation of nanomaterials. L939 fibroblasts and sp^2/0^ murine myeloma cells were used as samples for the in vitro cytotoxicity experiment. For the in vivo investigations, a naked mouse was used that had been injected with CQDs into the tail vein together with SMMC-7721 tumor cells [63]. When administered to cell samples in vivo and then in vitro, the CQDs generated from wheat straw and bamboo residues exhibit low toxicity, as can be seen. Through hydrothermal synthesis, Amit Kumar et al. investigated CQDs from Ocimum sanctum that successfully had good stability and displayed significant fluorescence [64]. When the triple-negative breast cancer cells (MDA-MB 478 cells) were exposed to the CQDs to detect Pb^2+^ ions, the CQDs showed low cytotoxicity and made for excellent fluorescent probes for multicolor cellular imaging. Another study suggested hydrothermally surface-modified CQDs for potential nanomedicine use, and cytotoxicity tests were carried out to gauge their biocompatibility. To observe cell proliferation, the cytotoxicity investigation on L929 murine fibroblasts was measured for 48 h [65]. The CQDs demonstrated non-cytotoxicity to the L929 mouse fibroblasts, which may penetrate cell membranes and emit fluorescence, staining cytoplasm, when different CQD doses were utilized.

### 3.4. pH Sensitivity

The pH sensitivity experiment makes it possible to determine how pH affects the optical properties of the sample. After adding a few drops of various pH solutions to the CQD sample, it will take some time for the sample’s emission to be visible. This type of result, which holds across a range of pH levels, is anticipated for CQDs with a sensitive pH. The properties of pH sensitivity can be used to detect changes in the pathological and physiological development of cells, inflammation, and muscular contraction. According to a study by Jayawera et al., a sample of durian shell CQDs was evaluated at various pH levels ranging from 3 to 11, and the PL intensity was consistent with two drops at pH 5 and pH 10. This is caused by the presence of the N-H and COO- groups on the surface of the CQDs, which resulted in a range of isoelectric points and dissociation constants [52]. Another study by Ding et al. demonstrated strong orange fluorescence CQDs by producing CQDs from phenylenediamine and ethane diamine using a straightforward microwave method [66]. It is noticed that the PL intensity increases as the pH decreases from 9.45 to 2.45 and that a linear detection zone with a progressive intensity shift in the pH range of 4.45 to 7 is present. CQDs with a high pH sensitivity can be beneficial for the early diagnosis of major illnesses such as cancer in vivo.

### 3.5. Quantum Yield

Fluorescence is the energy loss by a substance that has absorbed light by the emission of a photon, and the quantum yield is a measurement of the efficiency of photon emission through fluorescence. It is frequently described as the proportion of photons released to the absorbed photons. In other words, the fluorescence quantum yield estimates the likelihood that fluorescence would deactivate the excited state as opposed to other non-radiative mechanisms, such as internal conversion or vibrational relaxation, both of which result in non-radiative energy loss as heat to the environment. According to Jayawera et al., the QY for durian shell CQDs was calculated to be 12.93% using quinine sulfate [52]. According to another study, CQDs made from glucose using a hydrothermal technique yielded a QY in the range of 3.5% to 14% [65].

### 3.6. Additional Characterization of CQDs

#### 3.6.1. Moisture Retention Rate Test

According to a previous study, the moisture retention abilities of carbon CDs (Car-CDs) were further evaluated using an accepted methodology [67]. Car-CDs showed a dose-dependent trend in their ability to absorb moisture under various humidity conditions, and their ability to absorb moisture at high humidity levels was comparable to that of glycerin, a substance that is often utilized as a hygroscopic agent [68]. Similarly, Car-CDs retained moisture at rates of 78% and 84%, respectively, within 48 h at relative humidity levels of 43% and 81%. By enlisting ten volunteers to apply the Car-CD solution to their hands and compare the changes in moisture content before and two hours after the application, the moisturizing performance of Car-CDs on human skin was further assessed to investigate the moisture retention impact in vitro. Although the hand moisture content of the volunteers steadily decreased, the overall trend was constant, and the moisture improvement rate remained stable between 28% and 89%. Additionally, it was revealed that women have a moisturizing appreciation rate (MAR) much higher than men.

#### 3.6.2. Anti-Aging Test

According to research on anti-aging properties, the inhibition of aging-related enzymes is observed in both the presence and absence of CQDs. By using the 2-diphenyl-1-picrylhydrazyl (DPPH) radical scavenging assay and collagenase, tyrosinase, and elastase enzyme inhibition assays, anti-aging tests for polyphenol-derived bioactive CQDs were performed [69]. Son et al. also found that T-CQDs exhibited excellent inhibitory efficacy against collagenase, elastase, and tyrosinase, demonstrating antioxidant and anti-aging properties at 77.6 4.8%, 52.6 1.0%, and 44.21.3%, respectively [69]. At aging times of 0 h and 430 h, Hu et al. conducted an accelerated aging test using 800 W UV340 lamps [33]. As the exposure to UV radiation increased, the molecular chains of the PVA surface area were partially disrupted. However, the presence of T3-CQDs only causes point-to-surface protrusion, which indicates an improvement in the anti-aging properties of PVA.

## 4. Applications

The outcomes from the review highlighted the advantages of quantum dots (QDs) in health and beauty applications, such as anti-aging, lipstick, skin regeneration, angiogenesis, and antibacterial effects.

### 4.1. Health-Related Field

#### 4.1.1. Skin Regeneration and Wound Healing

Although CQDs were created using simple and affordable synthetic methods, their exceptional physicochemical and optical properties make them an ideal nanomaterial for use in the treatment of wounds. In vivo and in vitro tests have been used to illustrate the use of CQDs in wound healing. The majority of in vivo studies used wound healing assessments on the gross morphology of the wound, the size of the wound, and identification of the regenerated tissue based on histological evaluation to explore the biological effects of CQDs. According to one study, oxidative stress can significantly slow wound healing by releasing too many free radicals near the inflamed tissue. Carbonization-generated bioactive CQDs formed from polyphenols are capable of enhancing cell viability and promoting wound healing by stimulating fibroblast proliferation, accelerating cell migration, and reducing inflammation [70]. In addition, Zhao et al. asserted that the hydrothermal route for the synthesis of nitrogen-doped CQDs can be used to treat wounds infected with methicillin-resistant Staphylococcus aureus (MRSA) and to destroy the cell structure of *Staphylococcus aureus* with minimal side effects to the major organs of rats [71]. The CQDs in skin regeneration and wound healing are given in Table 2.

#### 4.1.2. Angiogenesis

Angiogenesis is a crucial process in wound healing that enables the transport of nutrients and gases to bodily tissues for cell proliferation and growth. A wound healing environment requires the physiological process of new blood vessel development. The characteristics of CQDs in angiogenesis can be observed based on the reaction of human umbilical vein endothelial cells (HUVECs) to the CQDs. According to Li et al., CQDs that use lysine and arginine as precursors efficiently promoted HUVEC growth with a high survival rate [10]. HUVECs were used in both live and dead assays. Using CQD urea, a different study examined the assays for measuring cell proliferation, in vitro tube formation, and in utero angiogenesis [81]. According to these findings, treatment of HUVECs with cytocompatible and hemocompatible CQD urea lengthens proliferation and improves proangiogenic responses in a dose-dependent manner. This study used the chorioallantoic membrane (CAM) method, which is widely used to examine angiogenesis, to test the angiogenesis response in an in utero chick. Due to its low cost, closed-system environment, good repeatability, and highly vascularized character, the CAM test is preferred. The effects of the CQDs on angiogenesis are shown in Table 3.

#### 4.1.3. Antibacterial Effect

Due to the higher occurrence of microbial infection, particularly in skin injuries, the complications of wound healing increase. The most frequently utilized microorganisms in antibacterial research are *Staphylococcus aureus* (*S. aureus*; Gram-positive) and *Escherichia coli* (*E. coli*; Gram-negative). A summary of the antibacterial effects of CQDs is shown in Table 4. To treat bacterial keratitis, carbon quantum dot polyamines (CQDPAs) have been created from polyamines via pyrolysis [84]. These tiny, highly cationic CQDs exhibited potent antibacterial properties and can damage bacterial membranes. According to Jian et al., they are good candidates for therapeutic applications in the treatment of bacteria-induced illnesses as well as eye infections. Zhao et al. tested quaternary ammonium CQDs made by a green approach utilizing 2,3-epoxypropyltrimethylammonium chloride and diallyl dimethylammonium chloride in mice in vivo and in vitro, and they displayed outstanding antibacterial efficacy against Gram-positive bacteria [85]. When treating Gram-positive bacterial infections, CQDs have an optimistic effect. According to Zhao et al., N-doped CQDs produced using hydrothermal synthesis show antibacterial effects on *Staphylococcus* and MRSA and have the power to break down their cell walls; however, they are ineffective against *E. coli*. Their mode of action illustrates how *Staphylococcus* germs connect to positively charged N-doped CQDs in certain places and interact with negatively charged bacteria. CQDs made from curcumin exhibited antiviral activity against enteroviruses, and the temperature at which they were heated during synthesis affected this activity [35]. The findings of this investigation demonstrated that they are highly biocompatible and have potent antiviral action in RD cells. *Staphylococcus aureus* germs are also resistant to CQD-antibacterial TiO_2_ effects [86]. As an antibacterial agent, CQD-TiO_2_ was added to bacterial cellulose (BC).

### 4.2. Cosmetic Field

#### 4.2.1. Anti-Aging and Sunscreen

Stress-related overexpression of collagenase, elastase, and tyrosinase and excessive sun exposure have been linked to various skin issues, including freckles, accelerated skin aging, wrinkle formation, and sunburn. Today, these issues may worry people. It has been demonstrated that the use of sunscreen helps protect the skin against UV damage. CQDs have compounds with good potential to meet the demand for new compounds to be utilized in sunscreens. This compound must have excellent UV absorption capabilities, photostability, and environmental friendliness. According to a study, CQDs made from citric acid using a hydrothermal approach and CQDs made from Dunaliella Salina using a carbonization process exhibit sun protection capability [94]. CQDs-DS were introduced to eukaryotic cells, resulting in minimal cytotoxicity while shielding the cells from UV light. Compared to CQDs made of citric acid, CQDs-DS have a better SPF. Tannic acid-derived CQDs (T-CQDs) have shown their ability to be CQDs with antioxidant and anti-aging characteristics by the pyrolysis synthesis technique [69]. T-CQDs demonstrated strong free radical scavenging potential and antioxidant and anti-aging activities against collagenase, elastase, tyrosinase, and skin aging-related enzymes. Moniruzzaman et al. found that carbonization-generated bioactive CQDs obtained from polyphenols can operate as an anti-aging oxidative stress attenuator [70], and CQDs with extensive polyphenolic polyaromatic domains and many -OH groups can greatly stop the aging of skin enzymes [69]. Citric acid with urea (T1-CQDs), citric acid with glycine (T2-CQDs), and citric acid with ethylene glycol (T3-CQDs) demonstrated a unique UV absorption range and claimed that the T3-CQDs achieved excellent UV absorption efficiency and good stability [33]. Hu et al. reported three types of CQD syntheses using a hydrothermal method. It has been demonstrated through human sunscreen experiments that the T3-CQDs have exceptional UV absorption characteristics to protect the skin and significantly extend service life by preventing UV radiation-induced polymer chain breaks. These experiments measured the number of erythema outbreaks and accelerated aging under UV radiation at 0 h and 430 h.

#### 4.2.2. Lipstick

**Figure 4:**
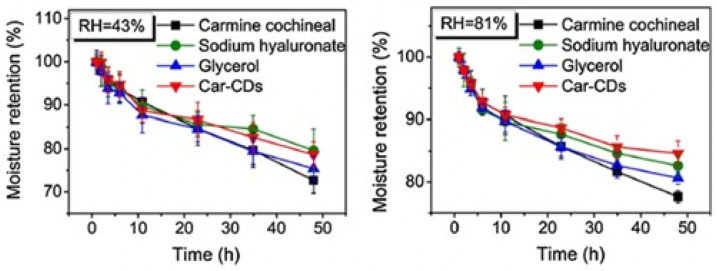
Moisture retention rate (MAR) at a relative humidity of 43% and 81%. Reproduced with permission from Ref [68]. Published by *Journal of Nanobiotechnology*, 2021.

CQDs are a superior substitute for other fluorescent nanomaterials because they are tiny fluorescent nanoparticles with high-moisture-retention capabilities. According to Cheng Dong et al., CQDs made from carmine cochineal as the raw material using a solvothermal process demonstrated good moisture retention performance, comparable to glycerin, which is often utilized as a hygroscopic agent [68]. At relative humidity levels of 43% and 81%, the moisture retention rates of the Car-CDs were 78% and 84%, respectively, after 48 h (Figure 4). Using the moisture measurement method, it has been determined that the use of Car-CDs as a nanoadditive in the creation of moisturizing lipsticks has further merit. The moisture (MAR) improvement rate was stable in the range of 27–89%. Compared with the three moisturizing solutions currently available on the market, the effectiveness of Car-CDs is much higher. The addition of Car-CDs has also dramatically increased by 13.94%.

## 5. Future Directions

CQDs have attracted significant interest in recent decades owing to their characteristics and possible use in various health and beauty-related disciplines. However, there is still an untapped potential for the development of CQDs. According to Alkhatib et al., activated charcoal can be utilized therapeutically and can absorb toxins and toxic compounds introduced into the stomach and intestine [92]. Charcoal Plus DS and CharcoCap are two popular names for dietary supplements used to absorb the gas produced by the stomach. Studies on CQDs as supplements or diarrhea remedies should be performed because of their hydrophilic nature and low toxicity. Due to their special qualities, such as biocompatibility, chemical stability, and adjustable PL, CQDs are currently exploited in pharmaceutical formulations and nanomedicine. Three gel formulations loaded with N-hydroxyphthalimide CQDs (NHF-CDs) were produced to assess their anticancer activities, according to Corina-lenuta et al. [95]. However, the impact of CQDs on peptide or protein fibrillation has not been thoroughly investigated. It has also been suggested that CQDs can prevent fibrillation of human insulin as a treatment method for disorders linked with peptide or protein fibrillation. Charcoal-based mouthwashes have seen a sharp rise in popularity in the commercial market, with claims of antibacterial action, anti-halitosis, caries reduction, and tooth whitening [96]. Research can be conducted on the potential of CQDs in pharmaceutical formulations, as there are still few studies available. Although several applications have been investigated, nothing is known regarding the use of CQDs as labels or tags. Surface passivation, customizable photoluminescence properties, and low toxicity make CQDs a better carrier for labels and tags than semiconductor QDs [9]. For instance, CQDs can be created that are long-term photostable, fluorescent, and non-photobleaching, and can stay in the skin to confirm immunization status. Due to the lack of infrastructure in underdeveloped countries, maintaining vaccination records can be challenging. To overcome this problem, a dissolvable microneedle patch can be used to inject CQDs under the skin and identify vaccination records using near-IR imaging. Individuals with allergies can use similar strategies. Doctors must offer first aid in the event of an accident, and checking for allergic reactions might indicate the difference between life and death before administering medications such as penicillin. Therefore, having CQD tags can help them recognize allergies.

## 6. Conclusions

The focus of this work was to highlight the existing situation and difficulties faced with the use of carbon quantum dots (CQDs) in wound healing and cosmetics. The synthesis and future course of this study were also explored. Different synthesis techniques have been examined for green chemistry, including top-down, bottom-up, waste-deprived, and functionalization techniques. CQDs are attractive materials because they are easy to use, non-toxic, affordable, straightforward, widely available, scalable, water-soluble, and chemically stable. CQDs can be engineered in different forms, sizes, functional groups, and colloidal stabilities. Functionalization plays a critical role, as the addition of groups such as amines and carboxyl can introduce surface modifications that significantly influence CQD behavior. Their optical, electrical, and toxicological properties have been investigated using techniques such as UV–Vis–NIR absorption, photoluminescence (PL) spectroscopy, and cytotoxicity assays. Strong UV absorption can be observed while fabricating CQDs using various synthesis methods; however, the peak UV absorption sites vary according to the method. According to the review findings, quantum dots (QDs) show promising potential in applications such as anti-aging, lipstick formulation, skin regeneration, angiogenesis, and antibacterial activity.

## Figures and Tables

**Table 1:** The advantages and disadvantages of the top-down approach and bottom-up approach.

Synthesis Method	Temperature Range	Thermal Characteristic	Advantages	Disadvantages
Hydrothermal	180–200 °C	Moderate, controlled heating in sealed reactor	Simple, cost-effective, high QY possible	Requires specific temperature optimization
Thermal Pyrolysis	High (varies by source)	Extreme heat in inert atmosphere	Scalable, inexpensive	Must carefully control to avoid over-oxidation
Microwave	Rapid heating	Short exposure to high energy	Fast, efficient	Less control over uniform heating
Arc Discharge	Extremely high	Electrical arc generates intense heat	High-energy production	Harsh conditions, expensive equipment
Laser Ablation	Localized extreme heat	Pulsed laser creates plasma state	Precise, efficient	Energy-consuming, expensive
Electrochemical	Room temp to 120 °C	Low to moderate temperature	Mild conditions, reproducible	May require longer processing time

**Table 2:** Study of CQDs in skin regeneration and wound healing.

Type of QDs	Synthesis Techniques	In Vivo Model	Results	Authors
CQDs	Data	Male rats 3–4 weeks old	CQDs are effective in improving cell viability and suppressing UV-induced ROS at the cellular level	[70]
NCQDs	Hydrothermal	Sprague–Dawley rats Weight 250 ± 20 g	The NCQD treatment group shows nearly complete healing after 14 days of treatment	[71]
Q-CQDs	Hydrothermal	Mice Weight 27 ± 2.5 g	The infected wounds almost completely recovered after ten days of treatment	[72]
GOQDs	Sonification	Rats 10–12 weeks old Weight 250–300 g	100% wound area closure after 16 days of treatment	[73]
CQDs	Microwave	Zebra fish group	CQDs with aloe vera accelerated the healing procedures and promoted restorative processes	[74]
CQDs	Hydrothermal	Sprague–Dawley rats Weight 279 ± 10 g	The treatment group recovered with healed wounds and new fur after 14 days of treatment	[75]
CQDs	Pyrolysis	Male mice 6–8 weeks old Weight 18–22 g	The CQD groups show complete healing within 5 days of treatment	[10]
ZnOCQDs	Sonification	Male mice	ZnO CQDs in the presence of GO-CS hydrogel promote 90% wound healing after 14 days	[76]
MoS_2_QDs	Sonification	BALB/c mice Eight weeks old	The treatment group shows almost 90% of injuries healed completely	[77]
VO_x_NDs	Hydrothermal	Sprague–Dawley rats 6 weeks old Weight 200 g	After six days, the H_2_O_2_/VO_x_NDs group promoted the highest wound healing by reducing the 60% wound area	[78]
GQDs	-	Male Wistar rats Weight 200–250 g	GQDs are able to accelerate the healing rate in burn injury	[79]
CQDs	-	Rats	Treatment with DFT-C/ZnO-hydrogel groups shows 95.7% wound closure after day 10 of treatment	[80]

**Table 3:** Effect of CQDs on angiogenesis.

Type of QDs	Synthesis Techniques	Experiment Model	Results	Authors
CQDs	Pyrolysis	HUVECs	Has a high survival rate with cell viability of more than 85%	[10]
CQDs	Hydrothermal	HUVECs	The proangiogenic response is shown in the HUVEC model	[81]
GQDs	-	HUVECs	The GQDs have a dose-dependent inhibitory effect on the proliferation, migration, tube formation, and sprouting of HUVECs	[82]
CQDs	Pyrolysis	Ovo chick	PLLCQDs proved to promote angiogenesis behavior	[83]

**Table 4:** Study of CQDs on antibacterial effect.

Type of QDs	Synthesis Techniques	Experiment Model	Results	Authors
CQDs	Pyrolysis	*E. coli * *S. aureus*	PLLCQD inhibition zone within 7–20 mm demonstrated an excellent antibacterial effect	[83]
CQDs	Pyrolysis	*E. coli * *S. aureus * *P. aeruginosa * *S. enteritidis * MRSA	The CQD_PAs_ demonstrated intense antibacterial activity	[84]
Q-CQDs	Hydrothermal	*E. coli * *S. aureus*	Q-CQDs inhibited the bacterial population in the wound area and reduced inflammation	[72]
CQDs	Hydrothermal	*E. coli * *S. aureus*	The CQD-TiO_2_ NPs show lower antibacterial activity against *E. coli* compared to *S. aureus*	[86]
CQDs	Hydrothermal	*E. coli * *L. monocytogenes*	The CQDs generate ROS, which eradicate 100% of the bacteria population	[87]
CQDs	Hydrothermal	*E. coli * *K. pneumoniae * *S. aureus * *L. monocytogenes*	The hCQDs effectively ruptured the bacterial membrane	[88]
CQDs	Hydrothermal	*B. subtilis * *E. coli * *S. epidermidis * *S. aureus * *S. dysenteriae * *S. paratyphi *-A serotype *P. aeruginosa * *S. pyogenes * *A. niger * *C. albicans*	*B. subtilis*, *E. coli*, *P. aeruginosa*, *S. pyogenes*, and *C. albicans* were highly sensitive to CQD NPs compared to others	[54]
CQDs	Hydrothermal	*E. coli*	The CQDs showed higher antibacterial activity in daylight compared to in the dark	[89]
CQDs	Hydrothermal	*E. coli * *S. aureus*	*E. coli* cell morphologies were ruptured, and *S. aureus* became irregular in the presence of P-doped CQDs	[90]
CQDs	Pyrolysis	*E. coli * *S. aureus * MRSA *C. albicans*	The production of ROS demonstrated high-efficiency antibacterial activity	[91]
CQDs	Hydrothermal	*E. coli * *L. monocytogenes*	The CQDs showed potent antibacterial activity against the bacteria	[92]
GOQDs	-	*E. coli * *S. aureus*	TA/KA-GOQDs have effective anti-microbial efficacy against bacteria	[65]
VO_x_NDs	Hydrothermal	*E. coli * MRSA	With the production of ROS, H_2_O_2_/VO_x_NDs are able to inhibit the growth of drug-resistant bacteria	[21]
CQDs	Pyrolysis	*E. coli * *S. aureus*	The cellular membrane is destroyed when exposed to the treatment, resulting in great antibacterial activity	[10]
CQDs	Hydrothermal	*B. cereus * *S. aureus * *P. aeruginosa * *V. cholerae * *E. coli*	The CQDs show high inhibitory activities for all bacteria tested, with an inhibition zone of 13–31 mm	[93]

## Data Availability

No new data were created or analyzed in this study.

## Abbreviations

The following abbreviations are used in this manuscript:

CQDs	Carbon Quantum Dots
QDs	Quantum Dots
CDs	Carbon Dots
SQDs	Semiconductor Quantum Dots
CNPs	Carbon Nanoparticles
PL	Photoluminescence
QY	Quantum Yield
LED	Light-Emitting Diode
GQDs	Graphene Quantum Dots
CNDs	Carbon Nanodots
PDs	Polymer Dots
CNTs	Carbon Nanotubes
NIR	Near-Infrared
UV	Ultraviolet Radiation
UV-Vis-NIR	Ultraviolet–Visible–Near-Infrared
HUVECs	Human Umbilical Vein Endothelial Cells
CAM	Chorioallantoic Membrane
CQDPAs	Carbon Quantum Dot Polyamines
QCE	Quantum Confinement Effect
Car-CD	Carmine Cochineal-Derived CD
DPPH	2-diphenyl-1-picrylhydrazyl
